# The separate and joint effects of recent interpersonal abuse and cannabis use on psychotic experiences: findings from students in higher education in the United States

**DOI:** 10.1007/s00127-023-02483-3

**Published:** 2023-04-24

**Authors:** Hans Oh, Jinyu Du, Nicole R. Karcher, Els van der Ven, Jordan E. DeVylder, Lee Smith, Ai Koyanagi

**Affiliations:** 1 Suzanne Dworak Peck School of Social Work, University of Southern California, Los Angeles, USA; 2 Southern Methodist University, Dallas, USA; 3 Department of Psychiatry, Washington University in St. Louis School of Medicine, St. Louis, USA; 4 Department of Clinical, Neuro- and Developmental Psychology, Vrije Universiteit Amsterdam, Amsterdam, Netherlands; 5 School of Social Service, Fordham University, New York, USA; 6 Centre for Health, Performance and Wellbeing, Anglia Ruskin University, Cambridge, UK; 7 Research and Development Unit, Parc Sanitari Sant Joan de Déu, CIBERSAM, ISCIII, Barcelona, Spain; 8 ICREA, Barcelona, Spain

**Keywords:** Psychosis, Cannabis, Interpersonal abuse, Adversity, Trauma

## Abstract

**Background:**

Various forms of interpersonal abuse (e.g., physical, emotional, sexual) and cannabis use across the lifespan have both been known to increase odds of psychotic experiences; however, there have been few studies examining their separate and joint effects in the United States.

**Methods:**

We analyzed data from the Healthy Minds Study (2020–2021) and used multivariable logistic regression and interaction contrast ratios to assess separate and joint effects of interpersonal abuse (past 12 months) and cannabis use (past 30 days) on psychotic experiences (past 12 months).

**Results:**

Students who only used cannabis had significantly greater odds of psychotic experiences (aOR: 1.70; 95% CI 1.58–1.82), as well as those who only experienced interpersonal abuse (aOR: 2.40; 95% CI 2.25–2.56). However, those who reported both cannabis use and interpersonal abuse had the greatest odds, exceeding the sum of these individual effects (the combined effect aOR: 3.46; 95% CI 3.19–3.76).

**Conclusions:**

Recent interpersonal abuse and recent cannabis use both separately and jointly increase odds of having recent psychotic experiences. Future research should continue to examine the potential interactive and additive impact of multiple known exposures to better inform primary and secondary prevention efforts.

## Introduction

Psychotic experiences are expressions of the psychosis phenotype that do not meet the clinical threshold for psychotic disorder and can affect upwards of 10% of the US general adult population [1–3], with varying prevalence depending on the measures and the populations [4, 5]. These psychotic experiences associate with a host of mental and physical health problems [6], as well as disability [7], suicidal thoughts and behaviors [8], and mortality [9]. Diathesis-stress models posit that genetic predisposition interacts with environmental factors to produce psychosis [10–12], including sub-threshold psychotic experiences in the general population [2]. Risk factors for psychotic experiences often mirror the developmental, cognitive, psychopathological, socio-environmental, and behavioral risk factors for schizophrenia [13]. Thus, as psychotic experiences gain public health significance, it is imperative to understand the risk factors for psychotic experiences and the conditions under which the risk factors may be particularly potent.

In this study, we focused on two major modifiable risk factors that have been examined in the literature, which are interpersonal abuse and cannabis use. In terms of interpersonal abuse, studies show that experiencing sexual, psychological, or physical abuse (especially during childhood) is associated with the greater risk for psychotic experiences [14], potentially by way of dissociation, emotion dysregulation, avoidance, hyperarousal, and negative schema [15]. Interpersonal abuse also activates the hypothalamic–pituitary–adrenal axis, which can dysregulate dopaminergic activity in the brain and give rise to hallucinations and delusions [16]. In terms of cannabis use, consistent evidence shows that cannabis use is also linked to psychosis [17–24], where psychotic illness is more common in people who use cannabis compared to those who do not use cannabis, that cannabis use and risk of developing psychotic illness have a dose-dependent relation, that people who use cannabis have an earlier onset of psychotic illness than people who do not use cannabis [18], and that cannabis use in young adulthood increase psychosis risk later in life [25, 26].

Emerging evidence suggests that the combined effects of interpersonal abuse and cannabis use may be particularly impactful. It is not uncommon for people to cope with interpersonal abuse using drugs (i.e., self-medication [27]). Further, substance use may also be a health behavior formed within a harmful or invalidating environment [28], whereby adverse social experiences sensitize and dysregulate activity of the mesolimbic dopamine system, increasing risk of psychosis [see the literature on social defeat; [29, 30]. Both factors can impact the dopaminergic system [31, 32], as interpersonal abuse can be traumatic and can elevate cortisol levels that associate with dopamine activity [16], while the active ingredient of cannabis (Δ9-tetrahydrocannabinol) can also mediate dopamine transmission [33]. It is possible that the factors operate via distinct mechanisms, and studies are showing that their joint effects may exceed their individual effects.

We have reason to suspect a synergy in joint effects drawing from studies on psychotic disorders. Houston and colleagues [32], for instance, found in a large sample of adults in the United Kingdom that history of childhood sexual abuse (before the age of 16) was more strongly associated with psychotic disorders among those who had ever used cannabis at any point in life (OR: 7.84; 95% CI 1.63–37.67) when compared to those who did not use cannabis (OR: 2.69; 95% CI 0.39–18.35). Similarly, Houston and colleagues [31] found in a large sample of adults in the United States that sexual trauma was more strongly and significantly associated with greater odds of psychotic disorders among people who had used cannabis before the age of 16 (OR: 11.96; 95% CI 2.10—68.22), than among those who did not use cannabis before the age of 16 (OR: 1.80; 95% CI 0.91–3.57). Sideli and colleagues [34] analyzed a sample of adults from South London, United Kingdom (231 individuals presenting for the first time to mental health services with psychotic disorders and 214 unaffected population controls) and found that while neither lifetime cannabis use nor history of childhood abuse was associated with psychotic disorder when included in the same model, their joint effect appeared to exceed the sum of their individual effects (OR: 2.94, 95% CI 1.44–6.02; ICR: 2.18, 95% CI 0.01–4.36) suggesting an additive interaction, though the interaction was not statistically significant after controlling for several covariates (ICR = 1.46, 95% CI  – 0.54 to 3.46).

However, there are fewer studies that have examined the synergies between interpersonal abuse and cannabis use with respect to psychotic experiences as the primary outcome. Harley and colleagues [35] found among a 211 adolescents aged 12–15 in Ireland that cannabis use (OR: 1.90; 95% CI 0.04–16.5) and childhood trauma (OR: 2.6; 95% CI 0.25–14.6) were significantly associated with greater odds of experiencing psychotic symptoms; however, when combined, the odds for psychotic symptoms exceeded either risk factor alone (OR: 20.90; 95% CI 2.30–173.50). Similarly, Morgan and colleagues [36] found in a large sample of adults in the United Kingdom that history of childhood abuse and cannabis use combined synergistically increased odds of psychotic experiences beyond the effects of each factor individually, though the interaction was only marginally significant.

These studies suggest that there may be greater than additive interaction between interpersonal abuse and cannabis use, but to our knowledge, there are no studies based in the United States that examine these synergistic effects of more recent forms of interpersonal abuse (i.e., over the past year) and cannabis use on recent psychotic experiences among young adults. Morgan and colleagues focused on childhood abuse but noted the potential for recent forms of interpersonal abuse to be especially impactful. Thus, in this study, we explore the research question: Do recent interpersonal abuse and cannabis use synergistically increase odds of psychotic experiences over the past year? We analyzed data collected from young adults aged 18–29 enrolled in 140 colleges/universities across the United States to explore this question.

## Methods

### Sample

We analyzed data from the 2020–2021 Healthy Minds Study (HMS), a non-probability web-based survey examining health and wellness among students enrolled in higher education in the United States [37]. The HMS survey is administered annually as a repeated cross-section of schools, with a different set of schools every year, including community colleges, four-year colleges, and professional schools. The HMS survey uses several validated measures to provide information about the prevalence of mental health outcomes, knowledge and attitudes about mental health, and service utilization. The survey was administered at 37 institutions of higher learning (*N* = 34,168) between September through December of 2020, and then administered again at 103 institutions (*N* = 103,748) between January through June 2021. The response rate was 14%, which is comparable to other response rates from online surveys using convenience samples and panels. We restricted the sample by age (18–29) to isolate young adults and further excluded individuals who were missing data on any of the variables of interest; we used complete-case analysis, resulting in a final analytic sample of 94,722. We used sample probability weights to adjust for non-response using administrative available data on full student populations at each institution, consistent with prior studies [37]. Using multivariable logistic regression, response propensity was estimated based on gender identity, race/ethnicity, academic level, and grade point average. We then assigned response propensity weights to each student who completed the survey. Students who were less likely to have completed the survey were assigned a larger weight in the analysis. Sample weights gave equal aggregate weight to each school in the national estimates rather than assigning weights in proportion to school size, so that overall national estimates were not dominated by schools in our sample with large enrollment. The HMS was approved by the Institutional Review Board Advarra, and the Institutional Review Boards at all participating campuses (IRB number: Pro00028565). Further, the secondary analysis presented in this study was deemed exempt under the approval of USC (UP-22–00068).

### Measures

*Psychotic experiences*. Psychotic experiences were measured using an abbreviated version of the World Health Organization Composite International Diagnostic Interview Psychosis Screen, which has been used in large global epidemiology studies [38]. Respondents were asked four questions about the following experiences: (1) A feeling something strange and unexplainable was going on that other people would find hard to believe; (2) A feeling that people were too interested in you or that there was a plot to harm you?; (3) A feeling that your thoughts were being directly interfered or controlled by another person, or your mind was being taken over by strange forces?; and (4) An experience of seeing visions or hearing voices that others could not see or hear when you were not half asleep, dreaming, or under the influence of alcohol or drugs? Respondents were then asked a single item (yes/no) about whether any of these four experiences occurred over the past 12 months. This variable was treated dichotomously in accordance with prior studies to signify the presence of psychotic experiences (i.e., hallucinatory experiences and/or delusional ideations) [38]. We focused on 12-month psychotic experiences to minimize recall bias.

*Interpersonal abuse*. Young adulthood abuse was measured using three dichotomous (yes/no) items: (1) Over the past 12 months, were you kicked, slapped, punched or otherwise physically mistreated by another person?; (2) Over the past 12 months, were you called names, yelled at, humiliated judged, threatened, coerced, or controlled by another person?; and (3) In the past 12 months, has anyone had unwanted sexual contact with you? (Please count any experience of unwanted sexual contact [e.g., touching of your sexual body parts, oral sex, anal sex, sexual intercourse, and penetration of your vagina or anus with a finger or object] that you did not consent to and did not want to happen regardless of where it happened). Interpersonal abuse was coded to reflect the presence of at least one of the three types of abuse.

*Cannabis use.* Cannabis use was measured using the item that asked respondents (yes/no) whether they had used marijuana over the past 30 days.

*Sociodemographic characteristics and mental health* (covariates)*.* We restricted the sample to focus on young adults further controlled for age as a continuous variable. We also adjusted for gender (cis-gender man, cis-gender woman, transgender/nonbinary/other), and race/ethnicity (White, Black, Latinx/Hispanic, Asian American/Pacific Islander, multiracial, and other). Education is a common proxy for socioeconomic status, and looking within a single socioeconomic stratum (i.e., students in higher education) can help isolate the effects of interpersonal abuse and cannabis use on psychotic experiences. While students in higher education represent a relatively high socioeconomic stratum, there is still a socioeconomic gradient within the stratum. Other common proxies (such as income and employment) may be inadequate measures of the gradient given that many students receive financial support from parents and do not have to work, and therefore do not earn any income. Food insecurity, however, has proven to be informative given that it is prevalent and a useful proxy for economic/financial instability [39], especially among young adults and college populations [40], and given its associations with mental health [41–43]. Food insecurity was assessed using two items, which asked: (1) Within the past 12 months I was worried whether our food would run out before we got money to buy more; (2) Within the past 12 months the food I bought just didn’t last and I didn’t have money to get more. Respondents could answer: never true, sometimes true, often true. Individuals were identified as food insecure with an affirmative answer (sometimes true or often true) to either question, following the two-item screen for families at risk of food insecurity [44]. We also adjusted for mental health using measures of depression and anxiety. Depression was measured using the Patient Health Questionnaire-9 (PHQ-9; Kroenke & Spitzer, 2002). The scale ranged from 0 to 27, which was dichotomized (scores 15 and higher) to reflect moderately severe to severe depression. *Anxiety* was measured using the General Anxiety Disorder – 7 (GAD-7; Spitzer et al. [46]). The scale ranged from 0 to 21 and was dichotomized (scores 11 and higher) to reflect moderately severe to severe anxiety [45, 46].

### Analysis

We calculated the prevalence of abuse, cannabis use, and all covariates (total and stratified by psychotic experiences). We opted to model predictions on an additive scale because multiplicative models assume that risks multiply in their effects, while additive models assume that psychosis can develop from either of the risk factors acting alone and synergistically (i.e., positive deviations from additivity). Additive models are particularly meaningful in psychiatric epidemiology because it is believed that psychiatric conditions generally have complex multifactorial etiologies. Following prior studies on the topic [35, 36], we tested for additive interaction [47], and depict the synergy between abuse and cannabis use by creating the following categorical variable: (1) no interpersonal abuse or cannabis use; (2) interpersonal abuse only; (3) cannabis use only; and (4) both interpersonal abuse and cannabis use. We adjusted for age, gender, race/ethnicity, food insecurity, depression, and anxiety. We calculated the interaction contrast ratio (ICR), which allows use of odds ratios derived from logistic models to estimate the relative excess risk resulting from the synergy of combined exposures (i.e., ICR = OR_interpersonal abuse & cannabis use_ — OR_interpersonal abuse only_ — OR_cannabis use only_ + 1). An ICR greater than zero signifies a positive deviation from additivity. We used nlcom command in Stata SE 15 to calculate confidence intervals and *p*-values for the ICR. To assess the potential influence of multicollinearity, we calculated the variance inflation factor (VIF) value for each independent variable. The highest VIF was 1.49, indicating that multicollinearity was unlikely to be a problem in our analyses [35].

## Results

The sample characteristics of the HMS have been detailed in prior studies. Table 1 provides the descriptive statistics and bivariate logistic regression models for all variables and their associations with psychotic experiences. Food insecurity and mental health problems (depression and anxiety) were more prevalent among people with psychotic experiences than among those without, and in unadjusted models, these factors were associated with significantly greater odds of psychotic experiences. Approximately 16.4% of the sample reported psychotic experiences over the past 12 months. Almost a third of the analytic sample reported any abuse over the past year, with emotional abuse being the most common, and physical abuse being the least. Any abuse was associated with 3.2-times greater odds of psychotic experiences. Various types of abuse varied between 2.77- and 3.2-times greater odds of endorsing psychotic experiences. The strongest associations were for emotional abuse. About one-in-five reported any cannabis use over the past 30 days, and cannabis use was associated with over double the odds of psychotic experiences.

**Table 1:** Descriptive statistics of analytic sample and bivariate logistic regression models predicting past-year psychotic experiences (Healthy Minds Study 2020–2021)

		Any psychotic experience(s) (*N* = 97,695)		
	Total *n* (%)	No (%)	Yes (%)	Unadjusted OR (*p*-value)
*Gender*				
Man	38,422 (39.33%)	32,248 (39.48%)	6174 (38.54%)	1.00
Woman	56,030 (57.35%)	47,284 (57.89%)	8746 (54.60%)	0.97 (0.91–1.02)
Trans/nonbinary/other	3243 (3.32%)	2145 (2.63%)	1098 (6.85%)	**2.67** (2.37–3.02)
*Race/ethnicity*				
White	59,458 (60.86%)	50,313 (61.60%)	9145 (57.09%)	1.00
Asian American/Pacific Islander	9098 (9.31%)	7726 (9.46%)	1372 (8.57%)	0.98 (0.87–1.10)
Black	10,624 (10.87%)	8680 (10.63%)	1944 (12.14%)	**1.23** (1.10–1.38)
Hispanic/Latinx	7719 (7.90%)	6321 (7.74%)	1398 (8.73%)	**1.22** (1.09–1.35)
Multiracial	9401 (9.62%)	7504 (9.19%)	1897 (11.84%)	**1.39** (1.28–1.51)
Other race	1395 (1.43%)	1133 (1.39%)	262 (1.64%)	**1.27** (1.02–1.59)
*Food insecurity (past 12 months)*				
Food secure	67,684 (69.28%)	58,675 (71.84%)	9009 (56.24%)	1.00
Food insecure	30,011 (30.72%)	23,002 (28.16%)	7009 (43.76%)	**1.98** (1.86–2.12)
*Depression (past 2 weeks)*				
No	75,177 (76.95%)	66,183 (81.03%)	8994 (56.15%)	1.00
Yes	22,518 (23.05%)	15,494 (18.97%)	7024 (43.85%)	**2.01** (1.89–2.13)
*Anxiety (past 2 weeks)*				
No	62,321 (63.79%)	55,909 (68.45%)	6412 (40.03%)	1.00
Yes	35,374 (36.21%)	25,768 (31.55%)	9606 (59.97%)	**3.34** (3.12–3.56)
*Abuse (past 12 months)*				
*Any abuse*				
No	65,483 (67.03%)	58,437 (71.55%)	7046 (43.99%)	1.00
Yes	32,212 (32.97%)	23,240 (28.45%)	8972 (56.01%)	**3.20** (3.04—3.37)
*Sexual abuse*				
No	90,157 (92.28%)	76,617 (93.80%)	13,540 (84.53%)	1.00
Yes	7538 (7.72%)	5060 (6.20%)	2478 (15.47%)	**2.77** (2.57—2.99)
*Physical abuse*				
No	91,126 (93.28%)	77,338 (94.69%)	13,788 (86.08%)	1.00
Yes	6569 (6.72%)	4339 (5.31%)	2230 (13.92%)	**2.88** (2.65–3.14)
*Emotional abuse*				
No	69,240 (70.87%)	61,442 (75.23%)	7798 (48.68%)	1.00
Yes	28,455 (29.13%)	20,235 (24.77%)	8220 (51.32%)	**3.20** (3.04–3.37)
*Cannabis use (past 30 days)*				
No	77,977 (79.82%)	66,886 (81.89%)	11,091 (69.24%)	1.00
Yes	19,718 (20.18%)	14,791 (18.11%)	4927 (30.76%)	**2.01 (1.89–2.13)**

*P* < 0.05 indicated in bold

**Fig. 1:**
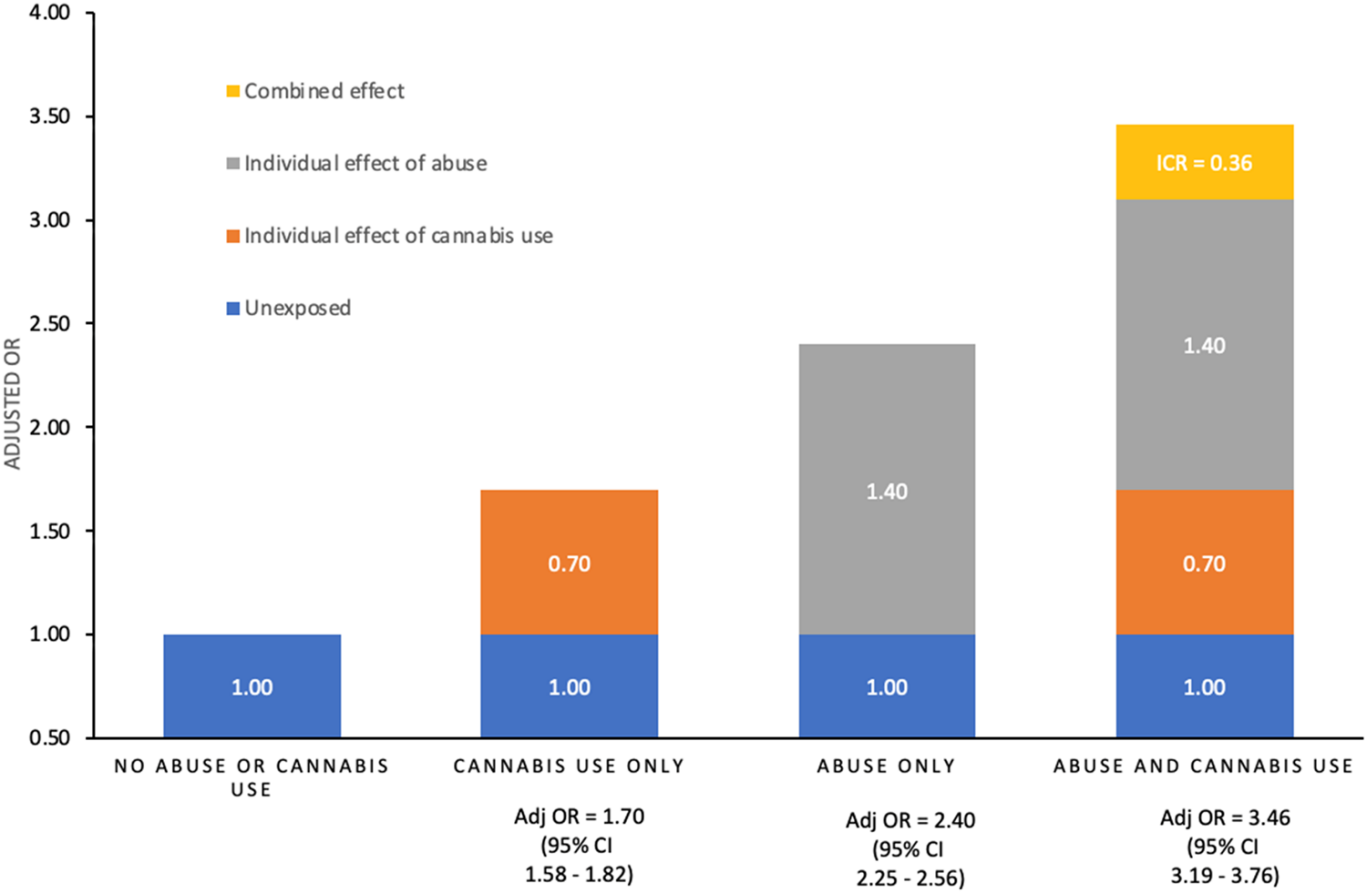
Separate and joint (synergistic) effects of interpersonal abuse and cannabis use on psychotic experiences among students in higher education (aged 18–29), Healthy Minds Study, 2020–2021 (*N* = 97,695). Abuse refers to interpersonal abuse over past 12 months. Cannabis use refers to any ‘marijuana’ use over the past 30 days. Psychotic experiences refer to any hallucinatory experiences or delusional ideations over the past 12 months. All models are adjusted for age, gender, race/ethnicity, depression, anxiety, and food insecurity

Figure 1 shows the synergistic effects of interpersonal abuse and cannabis use on odds of psychotic experiences on an additive scale. Those who only used cannabis had significantly greater odds of psychotic experiences (aOR: 1.70; 95% CI 1.58–1.82), and those who only experienced interpersonal abuse also had greater odds of psychotic experiences (aOR: 2.40; 95% CI 2.25–2.56); however, those who endorsed both cannabis use and interpersonal abuse had the greatest odds, exceeding sum of these individual effects (the combined effect aOR: 3.46; 95% CI 3.19–3.76). The ICR of 0.36 (95% CI 0.07–0.66; *p* = 0.015) on an additive scale indicates that the combined effect of interpersonal abuse and cannabis use is larger than the sum of the individual effects of the two exposures (i.e., 0.36 higher odds of psychotic experiences than if there were no synergy between interpersonal abuse and cannabis use).

## Discussion

### Main findings

In this study, we sought to extend previous research by examining the separate and joint effects of interpersonal abuse (emotional, physical, sexual, over the past year) and cannabis use (past 30 days) on odds of psychotic experiences (past year), adjusting for age, gender, race/ethnicity, food insecurity, anxiety, and depression. In terms of separate effects, we found evidence that interpersonal abuse and cannabis use were each associated with significantly greater odds of psychotic experiences. These findings comport with meta-analyses and systematic reviews showing the association between interpersonal abuse, cannabis use, and psychosis [14, 21, 22]. In terms of joint effects, we found evidence that exposure to interpersonal abuse and using cannabis together increased odds of psychotic experiences beyond either exposure individually. This aligns with a prior study among a general population sample of adults in the United Kingdom. Morgan and colleagues found that interpersonal abuse (OR: 2.04) and cannabis use (OR: 2.11) in the past year combined synergistically to increase odds of psychotic experiences (OR: 5.54), with an ICR of 2.40 (95% CI  – 0.17 to 4.97), though this was only marginally significant [36]. Our study found a smaller but statistically significant synergistic effect (*p* = 0.015). To our knowledge, our study was the first to test these main and synergistic effects in a large sample of students in higher education in the United States.

Our findings support and contribute to extant literature on psychotic experiences by examining the impact of environmental exposures and their synergies. Prior studies have focused on childhood adversities, since exposures that occur early in the developmental life course tend to shape later health outcomes [31, 32], and could set the trajectory for future exposures to risk factors, including revictimization [48] and cannabis use [49]. Our study examined recent exposures to interpersonal abuse, as studies have also noted recent interpersonal abuse can be particularly impactful on psychotic experiences [36]. The pathways by which interpersonal abuse and cannabis use interact to synergistically increase odds of psychotic experiences are not well understood. The separate and combined effects of interpersonal abuse and cannabis use align with social defeat [30] and other socio-developmental models [50]. It is possible that exposure to interpersonal abuse may lead to stress sensitization and cognitive biases, and in turn produce psychotic experiences [51]. Moreover, interpersonal abuse can be traumatic and lead to the formation of negative schemas that underlie hypervigilance and suspiciousness [52, 53], which can be further exacerbated by cannabis use [54].

### Limitations

Findings should be interpreted considering several limitations. First, in terms of design, the data were cross-sectional and did not allow us to establish the temporal order of events to make causal inferences. The relationships among interpersonal abuse, cannabis use, and psychotic experiences are difficult to disentangle; it is possible that interpersonal abuse can lead to cannabis use (as a form self-medication), in which case, cannabis could serve as a partial mediator, as indicated in prior studies [55, 56]. However, cannabis use may also occur before exposure to interpersonal abuse. And while socio-environmental exposures can precede psychotic experiences, studies also show that psychotic experiences can also occur throughout childhood before interpersonal abuse or cannabis use. Second, in terms of the sample, the study only examined students in higher education in the United States, and findings cannot be generalized beyond this population. The HMS employed a convenience sampling strategy that yielded a large sample but with a relatively low response rate (14%). The response rate is to be expected for online convenience samples [57, 58], and we attempted to account for non-response using survey weights, as done in prior studies using the dataset [37]. However, sampling bias remains a major concern. In terms of measurement, all measures used in the study failed to elicit adequate information about severity, frequency, and context of experiences. Notably the measures cover a relatively short timeframe. Further, the HMS asked whether individuals had used ‘marijuana’, and this item may not have captured all forms of cannabis use. Additionally, there may have been some social desirability bias in that students may have been reluctant to disclose interpersonal abuse, cannabis use, or psychotic experiences, given that the survey was administered through the institutions of higher learning in which the students were enrolled.

### Implications

Our findings show that the effects of interpersonal abuse and cannabis use are synergistic rather than overlapping, which may suggest the possibility that interpersonal abuse and cannabis use are linked to psychosis via different mechanisms. Moreover, socio-environmental risk factors for psychosis can combine synergistically to shape the expression and persistence of psychosis, potentially shaping one’s health trajectory toward need for care [59]. Since interpersonal abuse, cannabis use, and psychotic experiences may co-occur and coalesce over time, our study highlights the importance of examining the impact and interaction of multiple exposures and their underlying (and potentially distinct) mechanisms. While more research is needed, the co-occurrence of interpersonal abuse and cannabis use may nonetheless signal a higher clinical risk profile; prevention efforts may identify high-risk individuals and groups based on joint exposures and explore the utility of cannabis cessation support for people exposed to interpersonal abuse for psychosis prevention.

## Conclusion

Our findings provided evidence that interpersonal abuse and cannabis use both separately and synergistically increased odds of having psychotic experiences among students in higher education in the United States.
